# Experimental Evidence of Rainbow Trapping and Bloch Oscillations of Torsional Waves in Chirped Metallic Beams

**DOI:** 10.1038/s41598-018-37842-7

**Published:** 2019-02-12

**Authors:** A. Arreola-Lucas, G. Báez, F. Cervera, A. Climente, R. A. Méndez-Sánchez, J. Sánchez-Dehesa

**Affiliations:** 1 Wave Phenomena Group, Department of Electronic Engineering, Universitat Politécnica de Valencia, Camino de vera s.n. (Building 7F), ES-46022 Valencia, Spain; 2 Departamento de Física, Universidad Autónoma Metropolitana-Iztapalapa, Apartado Postal 55-534, 09340 Ciudad de México, Mexico; 3 Departamento de Ciencias Básicas, Universidad Autónoma Metropolitana-Azcapotzalco, Av. San Pablo 180, Col. Reynosa Tamaulipas, 02200 México Distrito Federal, Mexico; 4 Instituto de Ciencias Físicas, Universidad Nacional Autónoma de México, Apartado Postal 48-3, 62210 Cuernavaca Mor., Mexico

## Abstract

The Bloch oscillations (BO) and the rainbow trapping (RT) are two apparently unrelated phenomena, the former arising in solid state physics and the latter in metamaterials. A Bloch oscillation, on the one hand, is a counter-intuitive effect in which electrons start to oscillate in a crystalline structure when a static electric field is applied. This effect has been observed not only in solid state physics but also in optical and acoustical structured systems since a static electric field can be mimicked by a chirped structure. The RT, on the other hand, is a phenomenon in which the speed of a wave packet is slowed down in a dielectric structure; different colors then arrive to different depths within the structure thus separating the colors also in time. Here we show experimentally the emergence of both phenomena studying the propagation of torsional waves in chirped metallic beams. Experiments are performed in three aluminum beams in which different structures were machined: one periodic and two chirped. For the smaller value of the chirping parameter the wave packets, with different central frequencies, are back-scattered at different positions inside the corrugated beam; the packets with higher central frequencies being the ones with larger penetration depths. This behavior represents the mechanical analogue of the rainbow trapping effect. This phenomenon is the precursor of the mechanical Bloch oscillations, which are here demonstrated for a larger value of the chirping parameter. It is observed that the oscillatory behavior observed at small values of the chirp parameter is rectified according to the penetration length of the wave packet.

## Introduction

Structures in materials cause drastic effects in wave dynamics not only in quantum systems but also in macroscopic samples. In nanoscopic periodic structures, matter waves will be reflected or be able to cross the structure since in these kind of systems the spectrum became in allowed bands and gaps that permits or not the propagation in these ranges of energy, respectively. More complex structures yield other wave phenomena as the Bloch oscillations^1^, emerging in a crystalline structure in which a static electric field is applied, among many other quantum effects. Unfortunately, in quantum systems, the wave dynamics can hardly be measured and only its effects on transport properties, as for instance cross sections, can be seen.

In macroscopic structures other phenomena arise. This is the case of metamaterials^2,3^ in which a novel wave dynamics, that challenges intuition, can be obtained. Negative refraction, super-focusing, invisibility are some of these phenomena. Among them, the rainbow trapping effect is one of the most interesting phenomenon recently discovered in the field of optics^4^. In this effect the wave packets are slowed down up to different spatial depths, within a synthetic structure that has embedded a metamaterial with negative refractive index. Unfortunately the dynamics of all these phenomena cannot be easily observed in quantum mechanics since the construction of such structured materials is beyond the nanotechnology available today.

Mechanical waves have been converted in a golden vein. Several physical effects emerging in quantum and classical waves, that cannot be observed in other fields of physics, can be observed with vibrations: losses and decoherence do no affect strongly them. Opposite to other areas, there are not many experimental advances in elasticity: the control of elastic waves is mainly based on theoretical/numerical proposals^5^ though a few experiments have been already reported^6,7^. Vibrations usually involve a mixture of polarizations, which implies difficulties and technological challenges for measuring them selectively.

Here the emergence of two effects in torsional waves, the Bloch oscillations and the rainbow trapping, is shown. Furthermore, although the former was firstly found in quantum mechanics and the latter in metamaterials, a close relationship between both phenomena is here experimentally reported. Since the vibrations are important in the automotive, aerospace technology and principally in aeronautic industries, the results presented here could bring some light in the kind of structures needed to control them.

Electronic Bloch oscillations, on the one hand, are expected in solid state physics when a DC electric field is applied to a bulk semiconductor^1^. Due to intrinsic impurities, this quantum phenomenon was demonstrated in the late eighties thanks to the discovering of semiconductor superlattices^8^. The analogue of Bloch oscillations in classical waves has been also observed in dielectric structures^9^ and waveguides^10^, ultracold atoms^11^, phononic crystals^12,13^ and more recently in molecular motion^14^. Bloch oscillations are expected to occur on vibrating structures and experimental demonstrations of this effect for any of the different mechanical waves that can be propagated on them are of great interest.

The rainbow trapping effect, on the other hand, is an effect in which wave packets are slowed down up to different spatial depths, within a synthetic structure that has embedded a metamaterial with negative refractive index; the reached spatial depths depend on the central frequency of the wave packet. Since its discovery in 2007^4^, many potential applications have been reported^15–18^. For example, it will allow optical devices designed for the storage, processing and transmission of data to drastically increase their efficiency. A demonstration of its acoustic analogue has been reported by using acoustic metamaterials^19,20^ and sonic crystals^21,22^. More recently, the trapping of Lamb waves has been proposed theoretically^23^. An experimental realization for torsional waves is shown here.

## Results

### Frequency spectrum

**Figure 1:**
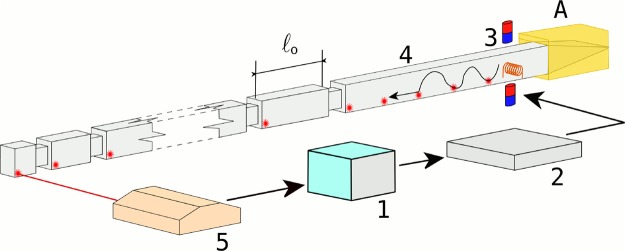
Scheme of the structured metallic beam and the experimental setup: (1) NI-PXI for generating, recording and analyzing signals, (2) high-fidelity audio amplifier, (3) electromagnetic-acoustic transducer (EMAT), (4) machined beam and (5) Doppler interferometer. The beam with rectangular section can be separated in three regions. On the right, a vibration isolation system (A) consisting of a wedge covered by an absorbing mastic seal. The central region is uniform while the region on the left contains the chirped structure. The lengths ${\ell }_{n}$ of the different cells *n* defining the structuration of the beam is determined by an analytical expression (see Eq. (1) in the text). The (red) spots indicate the position of the laser measurements.

As schematically illustrated in Fig. 1, the mechanical system under study consists on an metallic beam where three regions are clearly identified. At one end, a structured region has been machined. A uniform region follows, defining the central part of the beam, and the opposite end contains a passive vibration isolation system (A). The left-hand side of Fig. 1 shows the structured region of the beam, which is composed of cells with varying size ${\ell }_{n}$, defined between notches. A gradient of 20 cells whose size distribution mimics the effect of a DC electric field is used; the gradient defines the so called chirping parameter, *γ* of the resulting structure.

A given cell *n* of the chirped structure consists of a metallic block or cuboid with rectangular cross section of height *h* and width *w*. The corresponding block length ${\ell }_{n}$ is determined using the expression^24^

1 $${\ell }_{n}=\frac{{\ell }_{0}}{\mathrm{(1}+n\gamma )},$$

where *γ* is the dimensionless parameter and ${\ell }_{0}$ is a fixed arbitrary length defining the actual size of the series. The integer *n* takes values *n* = 0, 1, …, 19, defining the cells separated with notches. When *γ* = 0 a periodic structure is obtained while for *γ* ≠ 0 the structure becomes chirped and cells become smaller with increasing *n*. The *γ* parameter represents the analogous to a DC electric field and the construction rule used yields the Wannier-Stark ladders (WSL) for one-dimensional elastic systems^24^. In practice, the metallic beams are manufactured in aluminum using ${\ell }_{0}=92$ mm and the different cells are joined by small rectangular cuboids (notches) with the same dimensions: with height *h*_*C*_, width *w*_*C*_ and length ${\ell }_{C}$.

**Figure 2:**
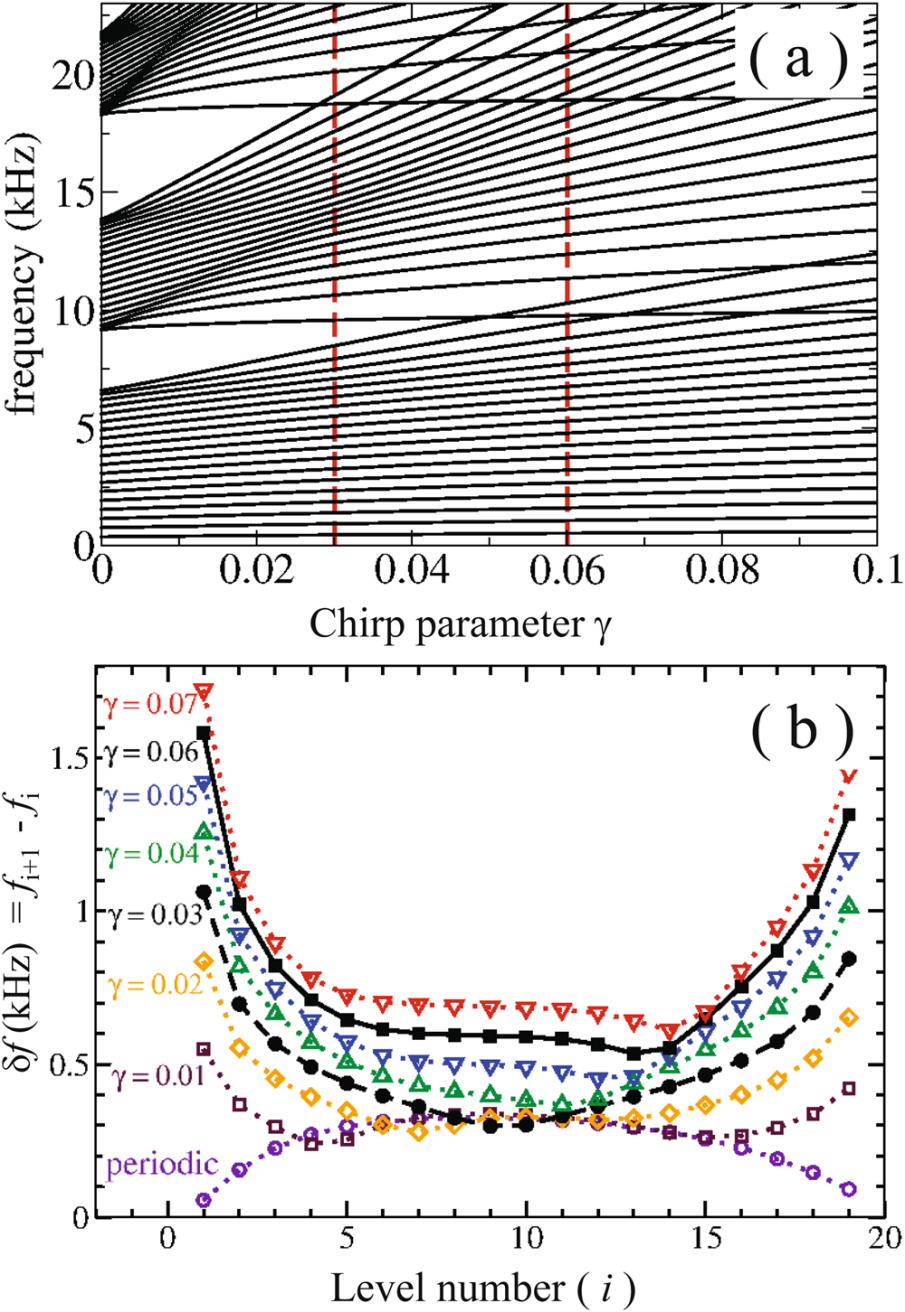
(**a**) Calculated band structure of the torsional modes propagating in a structured aluminum beam with rectangular cross section. The levels are given as function of the chirp intensity *γ*, a dimensionless parameter representing the elastic analogue of a DC electric field. The vertical dashed lines define the two chirped structures manufactured to demonstrate the Bloch oscillations and the rainbow trapping. They correspond to *γ* = 0.03 and 0.06, respectively. (**b**) Frequency interval, *δf*, between adjacent levels within the second pass band of the frequency spectrum represented in (**a**). The intervals are represented for several values of the chirp parameter *γ*; from *γ* = 0 (periodic) to 0.07. Notice that for *γ* > 0.03 the intervals between levels are approximately constant at the band center, defining the regime where Bloch oscillations can be observed.

Figure 2(a) reports the level dynamics calculated as function of the chirp parameter *γ*. The spectrum has been obtained using the transfer matrix (TM) method introduced by Morales *et al*.^25^ for beams with circular shape and here extended to beams with rectangular section^26^. The frequency levels are calculated assuming free boundary ends. The reader is addressed to the section Methods where we provide a brief account of the TM technique employed to calculate the one-dimensional spectrum of torsional waves propagating in structured rectangular beams. Notice that for *γ* = 0 a typical band structure, characteristic of a periodic system, emerges. In other words, a first band of levels starts at zero frequency and then bandgaps and passbands alternate in the frequency spectrum. The first bandgap is located approximately between 6.5 kHz and 9.5 kHz. The latter defines the beginning of the second band, which ends at around 13.5 kHz. For increasing values of *γ*, Fig. 2(a) shows that levels in a given band start to separate, the passbands become wider and the bandgaps narrower. In the second passband, three different regimes can be defined according to the behavior of the calculated level intervals δ*f*, which are shown in Fig. 2(b) for several values of *γ*. The first regime corresponds to values of *γ* ≠ 0, where the level density is inhomogeneous and has maxima close to the borders of the band; which is a reminiscence of the perfect periodic system. The second regime approximately corresponds to values 0.03 < *γ* ≤ 0.065, where the level density inside the band is approximately homogeneous. In other terms, the levels are almost equally spaced and the WSL dominates this regime^6,12^. It is shown that the intervals *δf* are almost constant in the middle of the band. Finally, the third regime is defined for larger *γ* values, when the levels of the nearest-neighbors bands strongly overlap with the second band, as it is shown in Fig. 2(a). Thus, after a certain critical value of the chirp parameter, which we has approximately established as 0.065, an enhanced transmission peak is expected to appear as a consequence of Zenner tunneling between bands^12^. Results presented here correspond to the first two regimes.

In order to characterize the regimes of interest here, we manufactured three different elastic structures, using *γ* = 0, 0.03, 0.06. The sample with *γ* = 0 contains a periodic structure consisting of 20 equal cells of length 92 mm and it is employed for comparison purposes. The samples with a *γ* parameter equal to 0.03 and 0.06 consist of 20 cells with variable lengths ${\ell }_{n}$ determined by Eq. (1). In what follows, it will be shown that wave packets of torsional waves propagating in this quasi one-dimensional elastic system exhibit the rainbow trapping effect and Bloch oscillations. The experimental characterization of these effects as as well as the connection between them are the main contributions of this work.

### Propagation of torsional waves in a periodic beam

**Figure 3:**
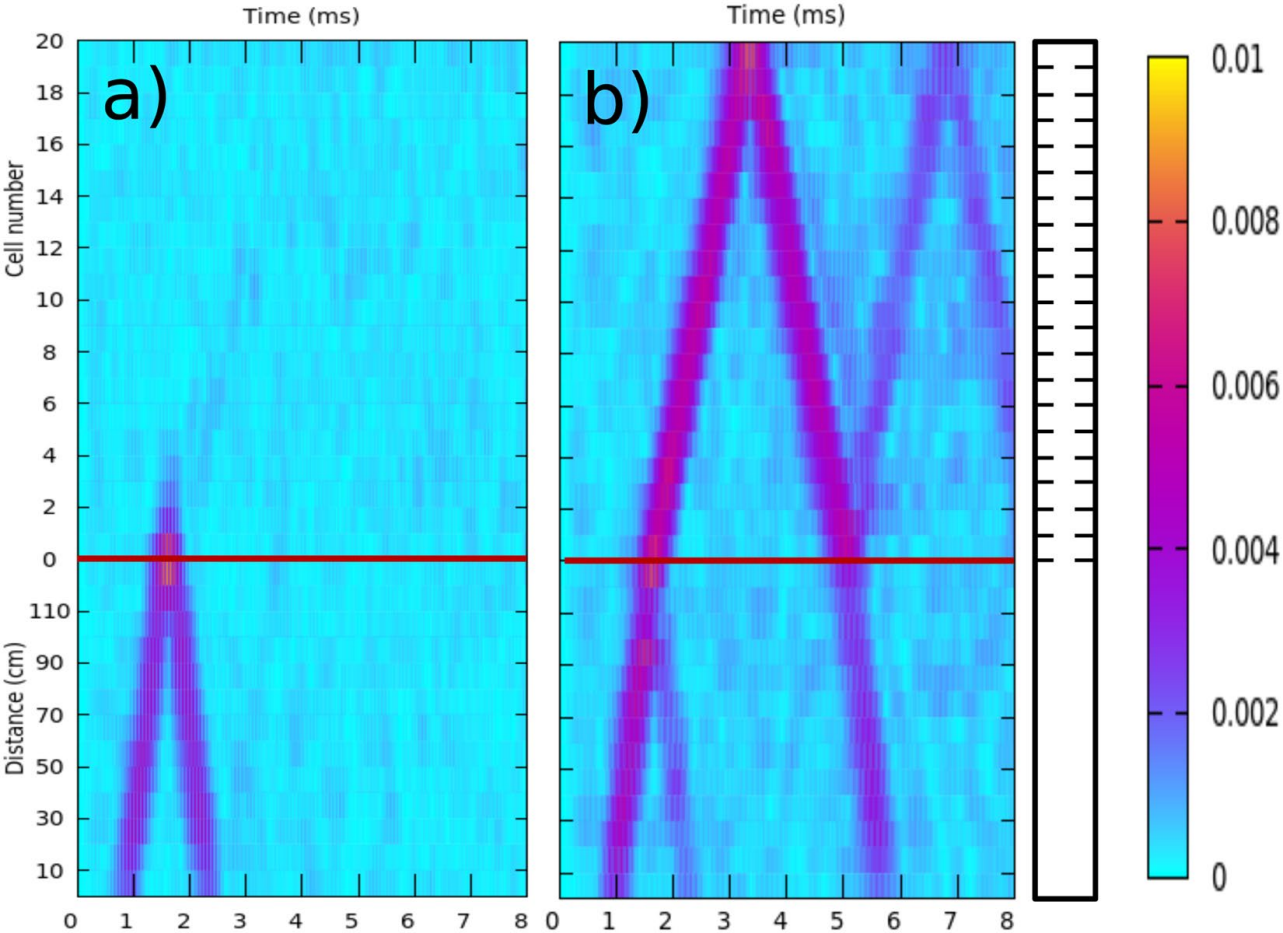
Measured time evolution of a wave packet of torsional waves propagating in the elastic beam containing a periodically structured region, corresponding to the sample *γ* = 0. (**a**) Results for a wave packet whose central frequency lies in the first gap (*f*_*C*_ = 8 kHz). It is observed that the wave packet is totally reflected at the interface. (**b**) Results when the central frequency lies in the second band (*f*_*C*_ = 11.5 kHz). Now, partial transmission and partial reflection of the packet is observed each time that the packet crosses the interface. The horizontal red lines are guides for the eye defining the interface between the uniform and the periodic parts of the beam. The beam is schematically drawn on the right-hand side. The color scale defines the measured amplitude of displacement (in arbitrary units).

The sample with *γ* = 0, corresponding to a locally periodic system, has been studied first for comparison purposes and for its own interest. The dynamics of wave-packets of torsional waves in this periodic structure is shown in Fig. 3. The time evolution of packets with central frequency, *f*_*C*_, within the first bandgap (*f*_*C*_ = 8 kHz) and inside the second passband (*f*_*C*_ = 11 kHz) are shown in Fig. 3(a,b), respectively. Both packets have an initial width of 0.5 ms. This width in the time domain implies that the original wave packet has a spatial extension of 0.875 m since the velocity of the torsional waves measured in a uniform beam is *c* = 1750 m/s.

On the one hand, it is observed in Fig. 3(a) that, when *f*_*C*_ belongs to the frequency gap, the wave packet is completely reflected at the interface with the periodically structured part of the beam. The horizontal red line, defining the interface between the uniform and the periodically structured parts of the beam, allows to distinguish an evanescent tail penetrating inside the periodic region. This behavior is usual for waves arriving at the interface with artificial structures having a Bragg bandgap.

On the other hand, Fig. 3(b) shows that the packet with *f*_*C*_ belonging to the second band is partially transmitted and partially reflected at the interface. Thus, each time that the packet crosses the interface this refraction phenomenon appears. It is also observed that the packet is totally reflected at the opposite end of the structure end because of the free boundary condition. In Fig. 3(b) we also observe two total reflections at the free end of the periodically structured part and two refractive phenomena at the interface between the uniform and structured parts of the beam.

In both Fig. 3(a,b) we observe no reflections coming from the end of the uniform part, demonstrating the total absorption of waves arriving at the dissipative end of the beam (see *A* in Fig. 1).

### Rainbow trapping

**Figure 4:**
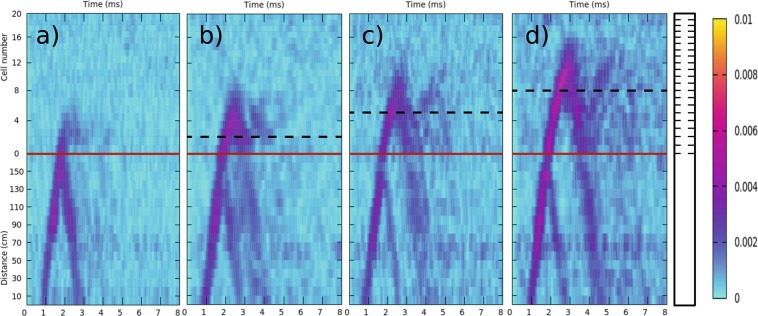
Experimental characterization of the rainbow trapping of torsional waves in a chirped aluminum beam. The propagation of a wave packet with central frequency, *f*_*C*_, is shown as a function of time and position in the sample with chirp intensity *γ* = 0.03. (**a**) Results for *f*_*C*_ = 9 kHz; (**b**) 10 kHz; (**c**) 11 kHz; and (**d**) 12 kHz. The beam is schematically drawn on the right-hand side. The color scale defines the measured amplitude of displacement (in arbitrary units).

The appearance of the mechanical rainbow trapping, here named *polyphonic trapping*, is investigated using the sample with smaller chirp intensity; i.e., *γ* = 0.03. The time evolution of wave packets with four different central frequencies are shown in Fig. 4(a–d), corresponding to frequencies *f*_*C*_ = 9 kHz, 10 kHz, 11 kHz and 12 kHz, respectively, inside the second band. It is observed how the wave-packet penetrates deeper inside the beam for increasing values of *f*_*C*_. This behavior provides the experimental demonstration of the *polyphonic trapping* of torsional waves, which is the mechanical analogue of the rainbow trapping effect in optics^4^. A similar behavior has been described in studying the transmission spectra of acoustic wave-packets propagating in chirped sonic crystals^22^.

**Figure 5:**
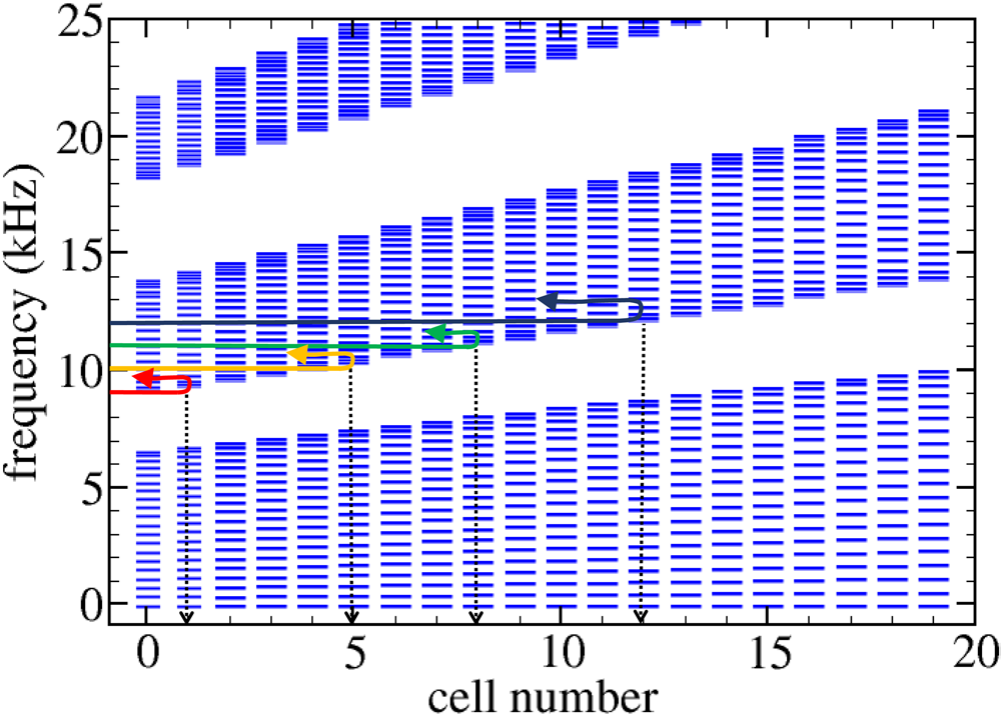
(**a**) Levels of the different minibands locally defined at the cells defining the structured beam with a chirp parameter *γ* = 0.03. White spaces between two consecutive set of levels define the local bandgaps. The colored lines describe qualitatively the behavior wave-packets centered at the frequencies analyzed in Fig. 4: 9 kHz (red), 10 kHz (yellow), 11 kHz (green) and 12 kHz (blue). The vertical arrows define the cells where the upper edge of the forbidden gap has the corresponding frequency, establishing the penetration lengths at which the wave-packets is reflected. Notice that wave-packets with higher frequencies are reflected at deeper distances inside the chirped beam, a feature defining the rainbow trapping effect.

The appearance of rainbow trapping in the beam with smaller chirping parameter intensity, *γ* = 0.03, can be explained by looking at Fig. 5, which represents the level spectra calculated at each cell of the chirped beam. These spectra have been obtained using the  transfer matrix method^25^ (see Methods). The elastic system can be considered as a gradient mechanical crystal in which there is a local variation of the bandgaps along the structure. For non-vanishing values of the chirping or gradient parameter the group velocity of the mechanical waves depends on the frequency and the position inside the beam. Therefore, the wave traveling inside this locally periodic crystal is gradually slowing down as the frequency of the propagating wave is approaching the “local” bandgaps^12^, where it is reflected. This behavior is observed in Fig. 5, where the transmitted bands (levels) and the bandgaps (white regions) are locally defined at each cell. The colored horizontal lines schematically describe the behavior of the different wave-packet characterized in Fig. 4. It is shown how the wave-packets with higher frequencies are reflected back at deeper distances inside the chirped beam; i.e., at the cell where the upper edge of the bangap coincides approximately with the central frequency of the packet.

Roughly speaking, the WSL associated to a given band *j* can be considered as a series of minibands whose lower band edges are determined by the condition $k{\ell }_{n}=j\pi$ for cell *n*, where *k* is the wavenumber and *j* = 1,2, … defines the order of the miniband. For the band of interest here, *j* = 1, the fundamental frequency *f*_0_ is determined with Eq. 1; i.e., ${f}_{0}=c\mathrm{/(2}{\ell }_{0})$. Then, it is possible to argue that a wave packet with a given *f*_*C*_ > *f*_0_ penetrates into the system up to cell *n* given by

2 $$n=\frac{1}{\gamma }({f}_{C}/{f}_{0}-\mathrm{1),}$$

which is derived using the assumption that ${f}_{C}\approx {f}_{n}=c\mathrm{/(2}{\ell }_{n})$. This equation yields the minimal penetration of the wave packet. The horizontal dashed lines in Fig. 4 define the cells *n* determining the minimum penetration of the corresponding wave-packet. in view of the simple model embedded in Eq. 2, the predicted values for *n* qualitatively describe the experimental observation; i.e., they show that the penetration depth of a wave-packet increases with its central frequency.

A better account of the cell where the wave-packet is fully reflected can be obtained graphically from the more exact calculation based on the TM method shown in Fig. 5. From this figure we conclude that the different packets penetrate till the cell (cuboid) number 1, 5, 8 and 12, respectively. Notice that this prediction is better but it is also approximate since deeper penetration distances inside the chirped region are observed (see Fig. 4) due to the evanescent behavior of waves with frequencies inside a bandgap.

### Mechanical Bloch oscillations

Let us describe now the emergence of the mechanical Bloch oscillations appearing in the characterization of the sample containing a chirped structure manufactured with *γ* = 0.06. This chirp intensity belongs to the second regime where the mean spacing of levels in frequency is approximately constant, as it is shown in Fig. 2(b). In spite of the frequency and position dependence of the torsional waves propagating in a chirped beam, the condition of a uniform level spacing is paramount in order to observe Bloch oscillations as it was discussed for acoustics and optical structures^9,12^. For *γ* = 0.06 this condition is accomplished for levels in the middle of the second band, as it is shown in Fig. 2(b).

For γ > 0 a simple model, (see Methods) indicates that the spacing *δf*_*B*_ between frequency levels is given by:

3 $$\delta {f}_{B}=\gamma \frac{c}{2{\ell }_{0}}\mathrm{.}$$

For the chirp intensity considered (6%), the predicted separation is *δf*_*B*_ ≈ 571 Hz, in good agreement with the separation calculated using the more exact TM technique, *δf*   ^(*TM*)^ = 600 Hz [see Fig. 2(b)].

**Figure 6:**
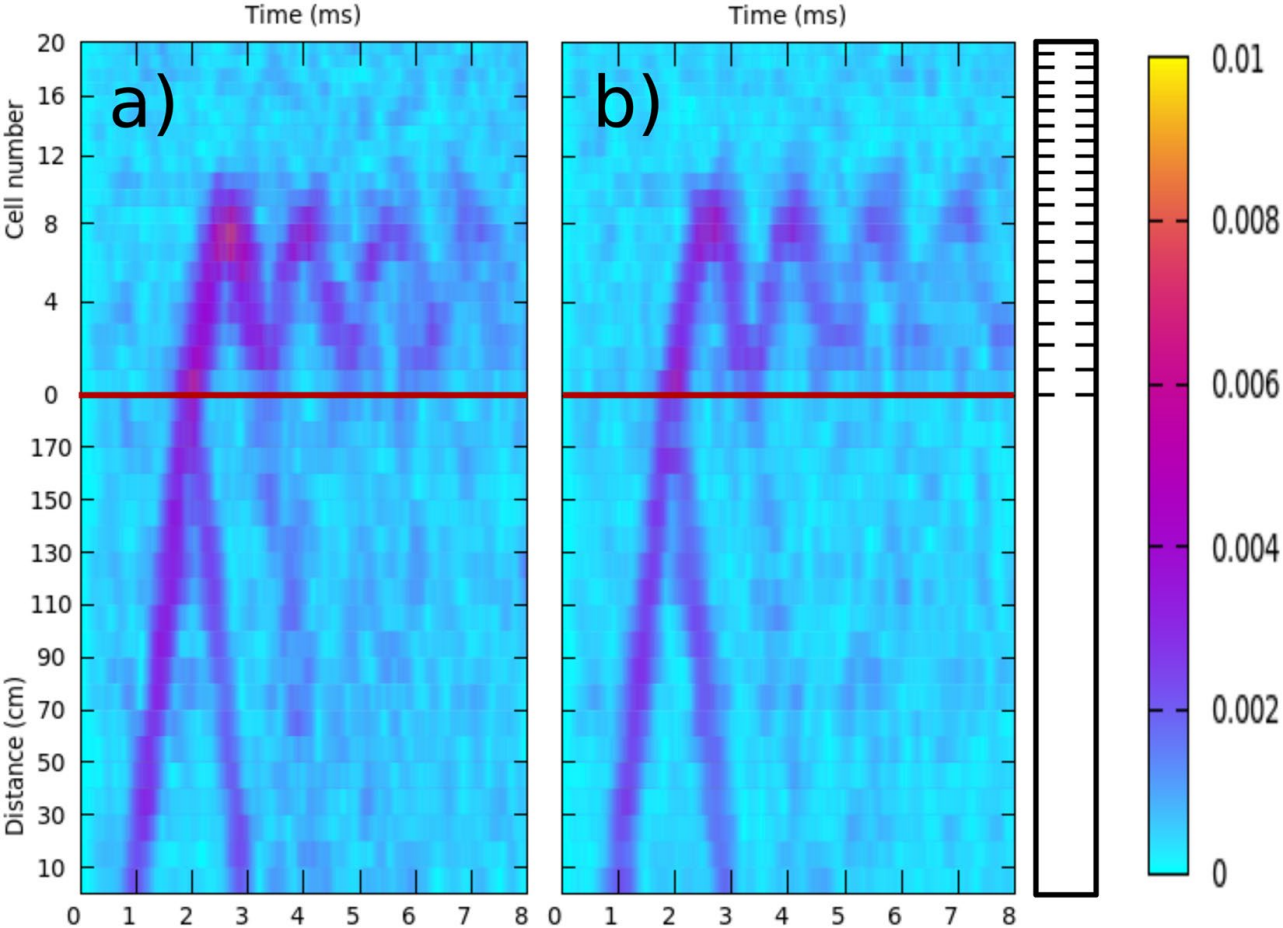
Experimental characterization of the Bloch oscillations of torsional waves propagating in a chirped structure with *γ* = 0.06. (**a**) Oscillatory behavior observed for a wave packet centered at *f*_*C*_ = 14.5 kHz. (**b**) Oscillations corresponding to a packet with *f*_*C*_ = 15 kHz. The horizontal red lines define the interface between the uniform and structured parts of the beam, which is schematically drawn on the right-hand side. The color scale defines the amplitude of displacement (in arbitrary units).

Experimentally, we have characterized the dynamics of wave packets with central frequencies belonging to the second band; from 10.5 kHz until 16 kHz with intervals of 0.5 kHz. The dynamics of the wave-packets corresponding to *f*_*C*_ = 14.5 kHz and 15 kHz are shown in Fig. 6(a,b), respectively. Both figures show an oscillatory behavior within the structured part of the beam with an equal oscillating period, *T*_*B*_, characteristic of the frequencies belonging to the middle of the band (see Fig. 2). These oscillations define the elastic version of the electronic Bloch oscillations. It is noticeable that Bloch oscillations for torsional waves are robust since four oscillations are clearly identified in Fig. 6 within the interval of 8 ms. The measured period ${T}_{B}(exp.\,\,)$ is about 1.6 ms, in good agreement with the predictions of the TM method ${T}_{B}(TM)=\frac{1}{\delta {f}_{B}(TM)}\approx 1.66\,{\rm{m}}{\rm{s}}$ and the simple model embedded in Eq. 3, for which ${T}_{B}(model)=\frac{1}{\delta {f}_{B}}\approx 1.75\,{\rm{m}}{\rm{s}}$.

**Figure 7:**
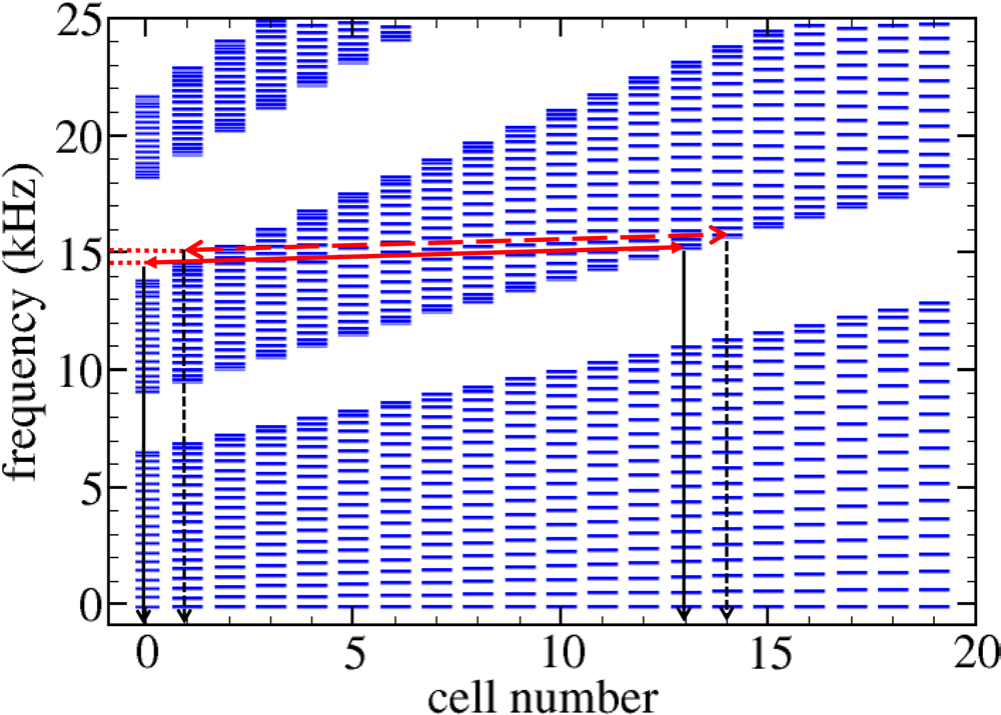
Levels of the different minibands locally defined at the cells of the structured beam with a parameter *γ* = 0.06. White spaces between two consecutive set of levels define the local bandgaps. The horizontal red lines defines the central frequencies of the wave-packets analyzed in Fig. 5: 14.5 kHz and 15 kHz. The arrows describes qualitatively the Bloch oscillations of the wave-packets. The packet centered at 14.5 kHz (continuous line) bounces between the cells defined by the vertical black arrows while that centered at 15 kHz (dashed line) does between cells defined by the dashed arrows. The red dotted lines indicates the leaking of energy through the interface with the uniform part of the beam as it is observed in Fig. 5. The amplitude of the oscillations for a given frequency is defined by the distance between vertical arrows.

The underlying physics behind Bloch oscillations can be understood by looking at the the local spectra represented in Fig. 7. For the two frequencies under study, defined by the horizontal lines in this figure, we can conclude that the corresponding wave packets penetrate inside the chirped region after crossing the local bandgaps existing at cells 0 and 1, where they experience a strong reflection. Inside the chirped region, the packet centered at 14.5 kHz oscillates between cells 13 and 0. For the packet centered at 15 kHz the oscillations take place between cells 14 and 1. Therefore, both oscillations have the same amplitude, which is determined by the distance between the maximum penetration inside the beam and the minimum distance to the interface with the uniform part. The amplitudes graphically derived from Fig. 7 are in qualitative agreement with the experimental ones shown in Fig. 6. In both panels of Fig. 6, it is also observed that each time that the wave packets approaches to the interface, a small portion of the energy of the wave packet leaks towards the uniform part of the beam. This leakage is a consequence of the evanescent behavior of waves arriving to the local bandgaps existing at the first cells of the chirped beam. This oscillatory effect works with transverse elastic waves (torsional) unlike the large number of devices proposed in the literature are strictly designed to control sound waves.

The Bloch oscillations are very well characterized by our experimental setup thanks to the high efficiency of the passive vibration isolation system, which fully absorbs all the arriving waves.

### Connection between rainbow trapping and Bloch oscillations

**Figure 8:**
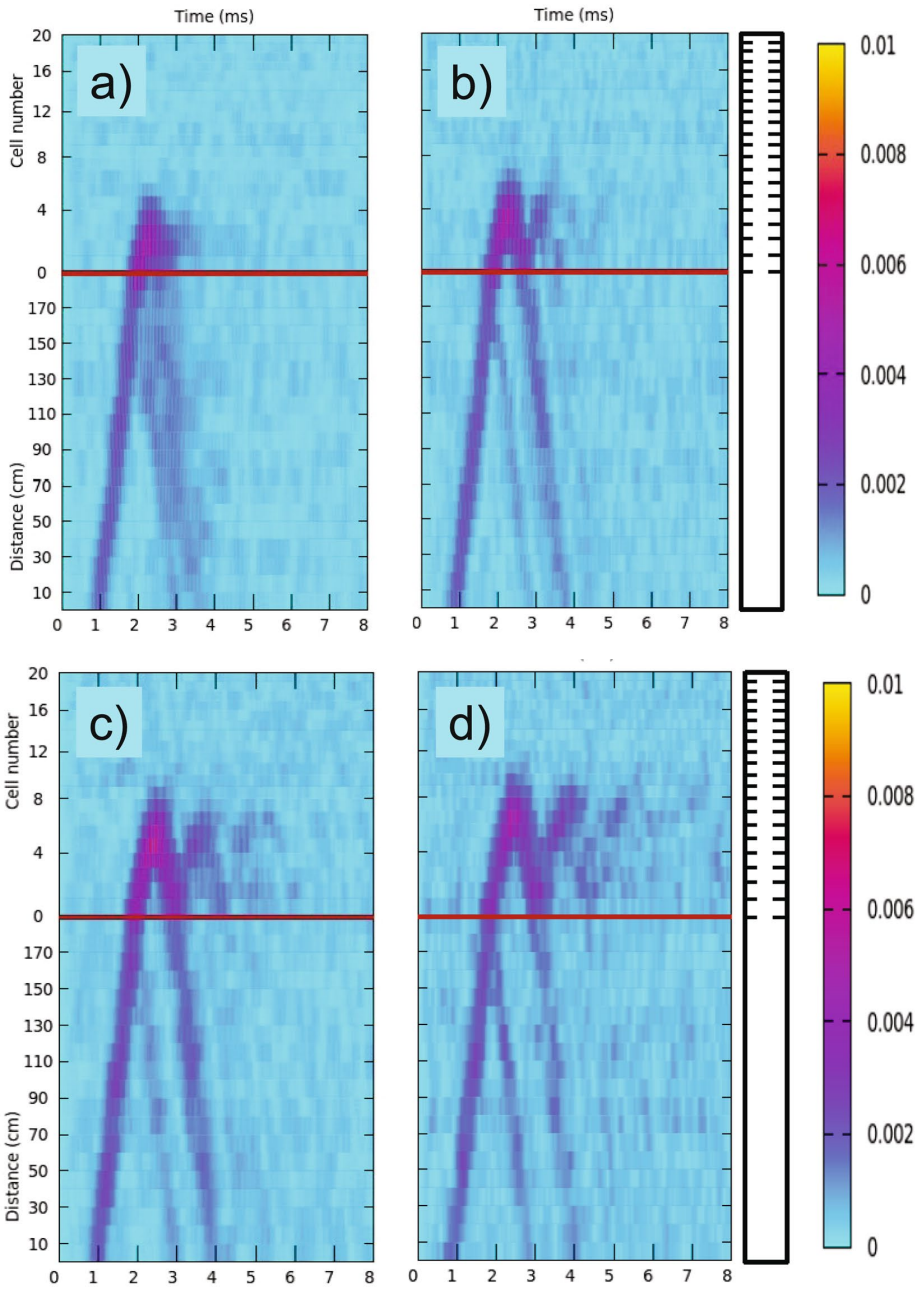
Experimental characterization of the *rectified rainbow-Bloch oscillations* in a chirped aluminum beam. The propagation of a wave packet with central frequency, *f*_*C*_, is shown as a function of time and position in the sample with chirp intensity *γ* = 0.06. (**a**) Results for *f*_*C*_ = 11 kHz; (**b**) 12 kHz; (**c**) 13 kHz; and (**d**) 14 kHz. The beam is schematically drawn on the right-hand side. The color scale defines the amplitude of displacement (in arbitrary units).

At this point it is interesting to remark the link observed between the rainbow trapping and the Bloch oscillations. In order to understand this relationship, Fig. 8 shows the dynamics of four different wave-packets propagating in the beam with chirping parameter *γ* = 0.06. The behavior of packets with central frequencies at 11 kHz, 12 kHz, 13 kHz and 14 kHz are given in Fig. 8(a–d), respectively. It is shown that for increasing values of *f*_*C*_ an oscillation emerges, which we associate to periodic reflections at the interface (horizontal red line), rather than with true Bloch oscillations. In fact, the actual Bloch oscillations appear at higher frequencies, as it has been previously discussed. The wave-packet reflected back by the rainbow trapping condition arrives to the interface, where it losses part of its energy. In other terms, it is partially transmitted to the uniform part and partially reflected back into the chirped region. As a result, the wave packet shows a *rectified* oscillation with a period that increases with frequency. This new kind of oscillation is here named as *rectified rainbow-Bloch oscillation* since its period increases with increasing the central frequency *f*_*C*_ of the wave-packet, as the rainbow trapping effect does for the penetration length. These oscillations are determined by two turning points: one is the rainbow trapping condition while the other is the interface with the uniform part of the beam. The period of the oscillations observed in Fig. 8(c,d) is increasing with the frequency up to 0.8 *T*_*B*_. The limiting case corresponds to the actual Bloch oscillations having a period *T*_*B*_. Thus, by increasing the frequency *f*_*C*_ to 14.5 kHz or 15 kHz we obtain that the interface is not anymore the turning point but a cuboid near the interface, as it is observed in Fig. 6. As a result, the strong energy leakage through the interface disappear and the true Bloch oscillations develop. Anyway, the turning point deep inside the chirped region is the same; that is, the rainbow trapping condition. Therefore, we can say that Bloch oscillations are triggered by the rainbow trapping condition. This connection between the two apparently different phenomena, rainbow trapping and Bloch oscillations, has not been discussed before and we expect to be general for all type of waves.

## Discussion

In summary, we have experimentally demonstrated the mechanical analogue of rainbow trapping and Bloch oscillations in one-dimensional elastic systems. Both effects have been extensively described in different structures supporting the propagation of electronic, photonic or acoustic waves but scarcely in elastic structures. On the one hand, the rainbow trapping was discovered for light waves propagating in photonic structures containing metamaterials with negative refractive index. On the other hand, the Bloch oscillations were characterized as a quantum phenomenon first characterized in semiconductors superlattices. We have shown the emergence of both phenomena by studying the propagation of torsional waves in chirped aluminium beams. Thus, wave-packets of torsional waves propagating in the chirped beam are reflected at different special depths according to its central frequency; larger penetrations correspond to higher frequencies. In addition, oscillations of a wave-packet are obtained when its central frequency is properly selected and the chirped beam is long enough. A non-trivial connection between both effects has been demonstrated: the rainbow trapping condition triggers the Bloch oscillations. When the structure is too small to hold Bloch oscillations, the wave-packet performs a new type of oscillation, here named *rectified rainbow-Bloch oscillations*, bouncing between two turning points defined by the rainbow trapping condition and the interface with the uniform part of the beam. The rectified oscillations have a period that increases with the central frequency of the wave-packet”.

The case of a non chirped beam, corresponding to an elastic periodic system with passbands and bandgaps has been also charcaterized for comparison purposes. The theoretical predictions of the independent rod model show a good agreement with experimental data. As a potential application we can foresee the control of torsional waves propagation in metallic rods, where their penetration length can be controlled using the rainbow trapping effect. Thus, the multiplexing of this type of mechanical signals seems possible.

## Methods

### Calculation of the frequency spectrum: The one-dimensional Transfer Matrix Method

In what follows, we present a brief description of the transfer matrix method developed to obtain the frequency spectrum reported in Fig. 2.

Lets consider a finite beam along the *z*-axis consisting of *M* rectangular blocks or cuboids, *C*_*i*_, whose dimensions change along the beam. Cuboid *i*, of width *w*_*i*_, height *h*_*i*_ and second moment of area *I*_*i*_, is located between positions *z*_*i*−1_ and *z*_*i*_ with *i* = 1,2, …, *M*.

The torsion in cuboid *i* is

4 $${\varphi }_{i}(z)={A}_{i}{e}^{{\rm{i}}k(z-{z}_{i-1})}+{B}_{i}{e}^{-{\rm{i}}k(z-{z}_{i-1})}\,{\rm{for}}\,{z}_{i-1} < z < {z}_{i}.$$

The continuity conditions for the torsion and the moment of torsion at *z* = *z*_*i*_ are

5 $${[{\varphi }_{i}]}_{{z}_{i}}={[{\varphi }_{i+1}]}_{{z}_{i}}$$

6 $${[{G}_{i}{\alpha }_{i}\frac{\partial {\varphi }_{i}}{\partial z}]}_{{z}_{i}}={[{G}_{i+1}{\alpha }_{i+1}\frac{\partial {\varphi }_{i+1}}{\partial z}]}_{{z}_{i}}$$

where *G*_*i*_ is the shear modulus of cuboid *i* and

7 $${\alpha }_{i}=\sum _{m=0}^{\infty }\sum _{p=0}^{\infty }\frac{\mathrm{256/}{\pi }^{6}}{{\mathrm{(2}m+\mathrm{1)}}^{2}{\mathrm{(2}p+\mathrm{1)}}^{2}}\frac{{h}_{i}{w}_{i}}{{(\frac{2m+1}{{h}_{i}})}^{2}+{(\frac{2p+1}{{w}_{i}})}^{2}}$$

is the Navier series^24^.

The amplitudes *A*_*i*+1_ and *B*_*i*+1_ are related to *A*_*i*_ and *B*_*i*_ through the transfer matrix

8 $$(\begin{array}{c}{A}_{i+1}\\ {B}_{i+1}\end{array})={{\mathbb{T}}}_{i\to i+1}(\begin{array}{c}{A}_{i}\\ {B}_{i}\end{array}),$$

where

9 $${{\mathbb{T}}}_{i\to i+1}=\frac{1}{2}(\begin{array}{cc}(1+\frac{{G}_{i}{\alpha }_{i}}{{G}_{i+1}{\alpha }_{i+1}}){e}^{{\rm{i}}k({z}_{i}-{z}_{i-1})} & (1-\frac{{G}_{i}{\alpha }_{i}}{{G}_{i+1}{\alpha }_{i+1}}){e}^{-{\rm{i}}k({z}_{i}-{z}_{i-1})}\\ (1-\frac{{G}_{i}{\alpha }_{i}}{{G}_{i+1}{\alpha }_{i+1}}){e}^{{\rm{i}}k({z}_{i}-{z}_{i-1})} & (1+\frac{{G}_{i}{\alpha }_{i}}{{G}_{i+1}{\alpha }_{i+1}}){e}^{-{\rm{i}}k({z}_{i}-{z}_{i-1})}\end{array}).$$

The amplitudes at the right end of the beam can be written in terms of the amplitudes of the left end as

10 $$(\begin{array}{c}{A}_{M}\\ {B}_{M}\end{array})={{\mathbb{T}}}_{M-1\to M}\,\cdots \,{{\mathbb{T}}}_{i\to i+1}\,\cdots \,{{\mathbb{T}}}_{1\to 2}(\begin{array}{c}{A}_{1}\\ {B}_{1}\end{array})={\mathbb{T}}(\begin{array}{c}{A}_{1}\\ {B}_{1}\end{array})\mathrm{.}$$

Free-free boundary conditions, used at both ends of the beam, imply

11 $${A}_{1}={B}_{1}\,{\rm{and}}$$

12 $${A}_{M}{e}^{{\rm{i}}k(L-{z}_{M-1})}={B}_{M}{e}^{-{\rm{i}}k(L-{z}_{M-1})}\mathrm{.}$$

After some algebra, the normal-mode frequencies of the structured beam are obtained finding the roots of the following equation

13 $$({{\mathbb{T}}}_{12}+{{\mathbb{T}}}_{11}){e}^{{\rm{i}}k(L-{z}_{M-1})}+({{\mathbb{T}}}_{22}+{{\mathbb{T}}}_{21}){e}^{-{\rm{i}}k(L-{z}_{M-1})}=0.$$

For the structures under study here, schematically represented in Fig. 1, the solutions of the last equation are shown in Fig. 2 as a function of the chirping parameter *γ*.

In the calculations, we have considered that the structures are composed of large cuboids, *W*_*i*_, with variable lengths ${\ell }_{{W}_{i}}$ and small cuboids or notches, *C*, with an equal short length ${\ell }_{C}=8$ mm. In addition, all the cuboids *W*_*i*_ have the same rectangular cross section, defined by the section of the aluminum beam (*w*_*i*_ = 10 mm, *h*_*i*_ = 30 mm and $\frac{{h}_{i}}{{w}_{i}}=3$); the total area being *S*_*W*_ = 300 mm^2^. However, the notches *C* have been manufactured with a much smaller rectangular cross section (*w*_*C*_ = 6 mm, *h*_*C*_ = 18 mm and $\frac{{h}_{C}}{{w}_{C}}=3$), that is *S*_*C*_ = 108 mm^2^.

In Fig. 2, the frequency levels corresponding to *γ* = 0 represents propagating waves in a beam where the length of the cuboids ${\ell }_{{W}_{i}}=$ constant. For parameters *γ* ≠ 0, the calculated frequency levels correspond to chirped beams in which the lengths of the cuboids *W*_*i*_ are determined by the expression given in Eq. 1.

### Band structure of a periodic beam

The dispersion equation for torsional waves propagating along the *z*−axis of a periodic structured beam consisting of alternating cuboids *W* and *C* with lengths ${\ell }_{W}$ and ${\ell }_{C}$, respectively, can be obtained using standard procedures. The cross sectional area of cuboids are *S*_*W*_ and *S*_*C*_, respectively and the lattice period is $\ell ={\ell }_{W}+{\ell }_{C}$.

Lets consider the general case of a two-component beam where the wavenumbers are ${k}_{i}=\frac{\omega }{{c}_{i}}$. The wave amplitudes for an unit cell of length $\ell$ can be expressed as:

14 $${\varphi }_{{\rm{L}}}=A{e}^{{\rm{i}}{k}_{C}z}+B{e}^{-{\rm{i}}{k}_{C}z}$$

15 $$\varphi =C{e}^{{\rm{i}}{k}_{W}z}+D{e}^{-{\rm{i}}{k}_{W}z}$$

16 $${\varphi }_{{\rm{R}}}=F{e}^{{\rm{i}}{k}_{C}z}+G{e}^{-{\rm{i}}{k}_{C}z}$$

where *ϕ*_L_, *ϕ*, and *ϕ*_R_ are the wave amplitudes for $x < -\,{\ell }_{W}/2$, $-\,{\ell }_{W}/2 < x < {\ell }_{W}/2$ and for ${\ell }_{W}/2 < x$, respectively.

Using the continuity conditions (5) and (6) at $z=-\,{\ell }_{W}/2$ together with the periodicity condition, we arrive to the following dispersion relation:

17 $$\cos ({k}_{Z}\ell )=\,\cos (\frac{\omega {\ell }_{C}}{{c}_{C}})\cos (\frac{\omega {\ell }_{W}}{{c}_{W}})-\frac{1}{2}(\frac{{G}_{C}{\alpha }_{C}}{{G}_{W}{\alpha }_{W}}+\frac{{G}_{W}{\alpha }_{W}}{{G}_{C}{\alpha }_{C}})\sin (\frac{\omega {\ell }_{C}}{{c}_{C}})\sin (\frac{\omega {\ell }_{W}}{{c}_{W}})$$

This expression is equivalent to that obtained for the propagation of acoustic waves in a two-component elastic superlattice^12^. The product *G*_*i*_*α*_*i*_ plays the same role than the acoustic impedance, *ρ*_*i*_*C*_*i*_, in the acoustic system.

In our single-component aluminum beam, *G*_*W*_ = *G*_*C*_ = *G* and *c*_*W*_ = *c*_*C*_ = *c*. since we are considering a single-component beam. In particular, the wave velocity for the torsional modes propagating in cuboids W and C, both with the same ratio $\frac{{h}_{i}}{{w}_{i}}=$ 3 is

18 $$c=0.55\sqrt{\frac{G}{\rho }},$$

where *ρ* is the beam density. For Aluminum, *c* ≈ 1755 m/s, very close to the measured value of 1750 m/s.

Regarding the Navier series *α*_*i*_, the lengths defining the transverse sections of cuboids are related through a uniform or isotropic scaling factor (*w*_*W*_ = *λw*_*C*_ and *h*_*W*_ = *λh*_*C*_), then *α*_*W*_ = *λ*^4^*α*_*C*_. This result is also valid for single-component period beams based on scaled circles, ellipses and triangles. In our samples, *λ* ≈ 1.67.

**Figure 9:**
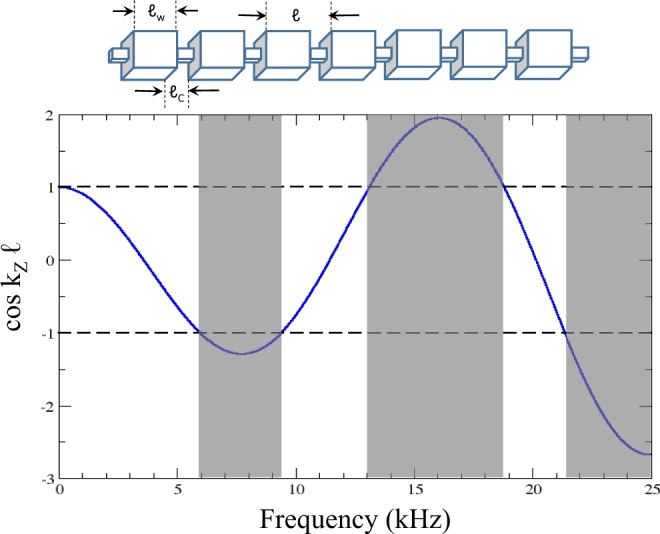
(Upper panel) Scheme of the chirped beam corresponding to *γ* = 0. It consists of a periodic distributions of large cuboids with length ${\ell }_{W}$ separated by small notches with a much smaller length ${\ell }_{C}$. The lattice period is $\ell ={\ell }_{W}+{\ell }_{C}$. (Bottom panel) Plot of the function $\cos ({k}_{Z}\ell )$ obtained from the analytical dispersion relation of torsional waves propagating in the periodic beam. The regions where $|\cos ({k}_{Z}\ell )| > 1$ define the bandgaps of the periodic beam.

Figure 9 plots a representation of the right-hand side of Eq. 17. Notice that frequencies where $|\cos ({k}_{Z}\ell )|\le 1$ define the passbands (white region). The rest of values are unphysical and define the bandgaps (grayed regions). The width of passbands and bandgaps obtained from this dispersion relation are in agreement with those calculated using the TM method (see the results at *γ* = 0 in Fig. 2).

### Frequency of Bloch oscillations: Model

The structured aluminum beam under study can be considered as a combination of 20 weakly interacting cuboids or elastic cavities *W*, each one *n* with different length, ${\ell }_{n}$, but with the same rectangular cross section *S*_*W*_. The cuboids *C* (notches) are the links between nearest-neighbors cavities *W*.

In this analytical model, the structure is considered as a set of 20 independent cuboids. For the cuboid with length ${\ell }_{n}$, the frequencies of the torsional normal modes ${f}_{j}^{(n)}$ are given by the expression^27^

19 $${f}_{j}^{(n)}=\frac{c}{2{\ell }_{n}}j,$$

where *j* is the number of nodes in the wave amplitude and ${\ell }_{n}={\ell }_{0}\mathrm{/(1}+n\gamma )$, with *γ* being the chirping parameter and ${\ell }_{0}=92$ mm.

- Results for the case *γ* = 0 (periodic structure)
  For this case the structure consists of alternating layers with fixed thicknesses ${\ell }_{W}=92$ mm, ${\ell }_{C}=8$ mm, and lattice period ${\ell }_{W}+{\ell }_{C}=100$ mm. We consider that cuboids *W* and *C* are weakly coupled and that there is a big mismatch between layer *C* and *W*; i.e., *G*_*W*_ *α*_*W*_ ≪ *G*_*C*_*α*_*C*_. The corresponding unperturbed (non-interacting) systems have their resonances centered at
  20 $${\omega }_{W}\approx \pi \frac{c}{{\ell }_{W}}j,$$
  for layers *W*, and for layers *C* at
  21 $${\omega }_{C}\approx \pi \frac{c}{{\ell }_{C}}i,$$
  where *i*, *j* are integers.
  Since $\mathrm{1/}{\ell }_{W}\ll \mathrm{1/}{\ell }_{C}$ one obtains that there is a narrow isolated tight-binding miniband around the second (*j* = 1) torsional mode (Fabry-Perot like resonance) in *W* layers. Thus, the dispersion relation of the miniband centered at $c/{\ell }_{W}$ takes the form:
  22 $$\omega \approx \pi \frac{c}{{\ell }_{W}}+B\,\cos ({k}_{Z}\ell ),$$
  where *B* defines the bandwidth.
- Results for *γ* ≠ 0 (non-periodic structures)
  For this case, the chirp parameter represents the driving force, which is the elastic analogue of the DC electric field in an electronic superlattice. The modes localized in the large metallic cavities ${\ell }_{n}$ depend approximately linearly on the driving force ∂*ω*/∂*Z* with the slope given by (for *j* = 1 in Eq. 19)
  23 $$\partial \omega /\partial Z=\pi c\partial /\partial Z\mathrm{(1/}{\ell }_{W})$$
  For the case considered (*j* = 1), a linear variation of frequency is obtained by introducing a constant variation $1/{\ell }_{W}$:
  24 $$\delta \omega =\omega ({\ell }_{n})-\omega ({\ell }_{n-1})=\pi c\delta \mathrm{(1/}{\ell }_{W})$$
  Where
  25 $$\delta \mathrm{(1/}{\ell }_{W})=\mathrm{1/}{\ell }_{n}-\mathrm{1/}{\ell }_{n-1}=\gamma /{\ell }_{0}\mathrm{.}$$
  The expression above has been obtained by using the thickness variation ${\ell }_{n}={\ell }_{0}\mathrm{/(1}+n\gamma )$, with *γ* = const. Therefore, such thickness variation supports the formation of WSL. The spacing between levels is determined by
  26 $$\delta {\omega }_{B}=2\pi \delta {f}_{B}=\omega ({\ell }_{0})\gamma =\gamma \frac{\pi c}{{\ell }_{0}},$$
  where ${\ell }_{0}$ is a fixed length, in our case ${\ell }_{0}={\ell }_{W}=9.2$ cm.
  A system with a discrete sequence of frequency levels with level spacing *δω*_*B*_ is the elastic equivalent of the electronic WSL, and it is expected to exhibit elastic oscillations with period $T=\frac{2\pi }{\delta {\omega }_{B}}$, as it has been experimentally observed for *γ* = 0.03 and *γ* = 0.06 in Figs 4 and 5, respectively of the manuscript.
- Case *γ* = 0.06
  Using the previous formula, we obtain that the spacing between linear frequencies is *δf*_*B*_ = 571 Hz and the corresponding Bloch oscillation period is *T*_*B*_(model) = 1.7 ms. In view of the simplified model employed, these values are in relative good agreement with the ones obtained using the TM method and with those experimentally reported in Fig. 4. Thus, for the oscillation period *T*_*B*_(TM) = 1.66 ms and *T*_*B*_(exp.) = 1.6 ms.
- Case *γ* = 0.03

For this value is *f*_*B*_ = 285 Hz and the Bloch period is *T*_*B*_ = 3.5 ms

### Experimental characterization

The three manufactured metallic beams were experimentally characterized using Doppler interferometry. The time evolution of a torsional wavepacket has been obtained inside and outside the chirped structure in the beam. First, torsional waves are generated in the uniform part of the beam and sent to the chirped structure using the experimental setup schematically depicted in Fig. 1. The signal generated by a NI-PXI (1) is amplified by a high-fidelity audio amplifier Cerwin-Vega CV5000 (2) and then sent to (3), an electromagnetic-acoustic transducer (EMAT). The EMAT generates a torsional Gaussian wave packet at its position, located in the uniform part of the beam. The wave packet travels towards the chirped structure and the vibrations are measured with a laser Doppler vibrometer (5) and analyzed with the PXI. In order to reconstruct the dynamics of the wave packets, the measurements are done in several positions along the structured part as well as in the uniform part of the beam. For frequencies higher than ≈1.5 kHz, the waves arriving to the opposite side of the structured beam are almost completely absorbed by a passive vibration isolation system (A). The latter consists of a wedge covered by an absorbing mastic seal.

## Supplementary information

LaTeX Supplementary File
