# Supplementary figures and images for: Experimental Evidence of Rainbow Trapping and Bloch Oscillations of Torsional Waves in Chirped Metallic Beams
Source: Sci Rep. 2019 Feb 12;9:1860. doi: 10.1038/s41598-018-37842-7 (PMC6372627; doi:10.1038/s41598-018-37842-7)

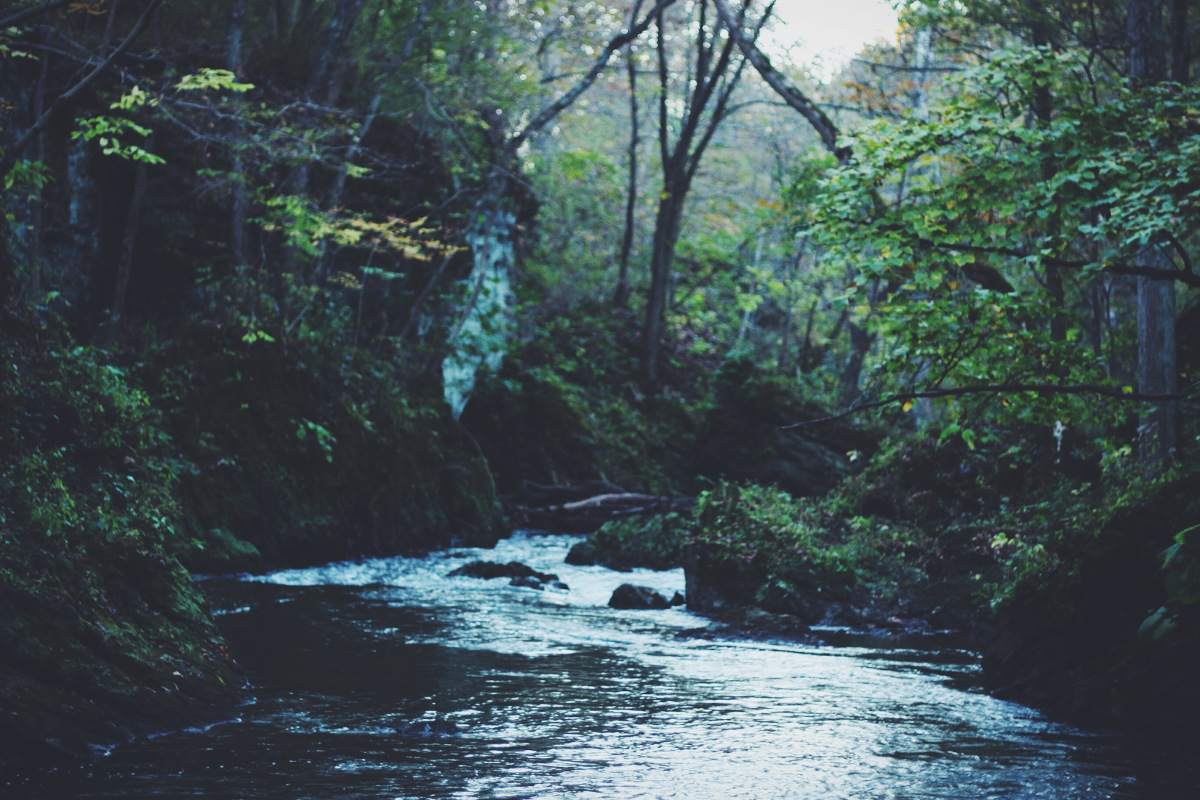

Supplement: Supplementary file 1 — LaTeX Supplementary File [file 41598_2018_37842_MOESM1_ESM.jpg]
